# Successful treatment of remnant gastric cancer with afferent loop syndrome managed by percutaneous transhepatic cholangial drainage followed by elective gastrectomy: a case report

**DOI:** 10.1186/s40792-021-01304-6

**Published:** 2021-09-28

**Authors:** Shu Aoyama, Masaaki Motoori, Yasuhiro Miyazaki, Tomoki Sugimoto, Yujiro Nishizawa, Hisateru Komatsu, Akira Inoue, Yoshinori Kagawa, Akira Tomokuni, Kazuhiro Iwase, Kazumasa Fujitani

**Affiliations:** 1 Department of Gastroenterological Surgery, Osaka General Medical Center, 3-1-56 Bandaihigashi Sumiyoshi-Ku, Osaka, Japan; 2 Department of Gastroenterological Surgery, Graduate School of Medicine, Osaka University, 2-2 Yamadaoka, Suita-shi, Osaka, Japan

**Keywords:** Afferent loop syndrome, Remnant gastric cancer, Percutaneous transhepatic cholangial drainage

## Abstract

**Background:**

There are only few reported cases of remnant gastric cancer with concomitant afferent loop syndrome. Emergency surgery is the standard treatment strategy for this disease. However, some afferent loop syndrome cases, especially those with complete obstruction, can lead to a septic state, which makes performing emergency surgery risky. We describe a case of remnant gastric cancer with complete afferent loop obstruction, which was successfully managed by radical surgery following percutaneous transhepatic cholangial drainage of the afferent loop.

**Case presentation:**

A 71-year-old man presented with nausea and abdominal discomfort. When he was 27 years old, he had undergone distal gastrectomy for a benign gastric ulcer, with gastrojejunostomy (Billroth II reconstruction). Abdominal computed tomography revealed thickening of the anastomosis site and significant dilation of the afferent loop. Gastrointestinal fiberscopy revealed advanced remnant gastric cancer at the anastomosis site, and the stoma of the afferent loop was completely obstructed. We diagnosed the patient with remnant gastric cancer with afferent loop syndrome. Percutaneous transhepatic cholangial drainage was performed twice before surgery to decompress the afferent loop. This provided more time for the patient to recover. Radical surgery of total remnant gastrectomy and Roux-en-Y reconstruction were performed electively. There were no severe postoperative complications. The patient died 8 months following the operation owing to peritoneal dissemination recurrence.

**Conclusion:**

We encountered a case of remnant gastric cancer with afferent loop obstruction, which was successfully managed by radical surgery following decompression of the afferent loop by percutaneous transhepatic cholangial drainage. Percutaneous transhepatic cholangial drainage effectively managed the afferent loop syndrome, resulting in the safe performance of elective surgery.

## Background

Remnant gastric cancer is defined as gastric cancer arising in the remnant stomach following partial gastrectomy for a benign or malignant disease [1]. The incidence of remnant gastric cancer is reportedly 2.6% [2], and remnant gastric cancer is frequently diagnosed at an advanced stage with a lower chance of cure than primary gastric cancer [3]. There have been few reported cases of remnant gastric cancer with concomitant afferent loop syndrome. The standard treatment strategy for afferent loop syndrome secondary to any cause is emergency surgery.

However, some afferent loop syndrome cases, especially those with complete obstruction, can lead to a septic state owing to severe pancreatitis, cholangitis, bowel necrosis, or perforation [4]. In addition, patients with afferent loop syndrome tend to have poor nutrition due to symptoms such as vomiting, nausea, and abdominal discomfort [5]. Therefore, emergency surgery for remnant gastric cancer with afferent loop syndrome carries significant additional risks.

We report a case of remnant gastric cancer with complete afferent loop obstruction, treated with elective and radical surgery after controlling the afferent loop syndrome by percutaneous transhepatic cholangial drainage (PTCD).

## Case presentation

A 71-year-old Japanese man presented with nausea, upper abdomen discomfort, and weight loss of 10% during the past year (62.9 kg at presentation; the former body weight was 70 kg). He had a surgical history of distal gastrectomy with Billroth II reconstruction for a benign gastric ulcer when he was 27 years old.

**Fig. 1:**
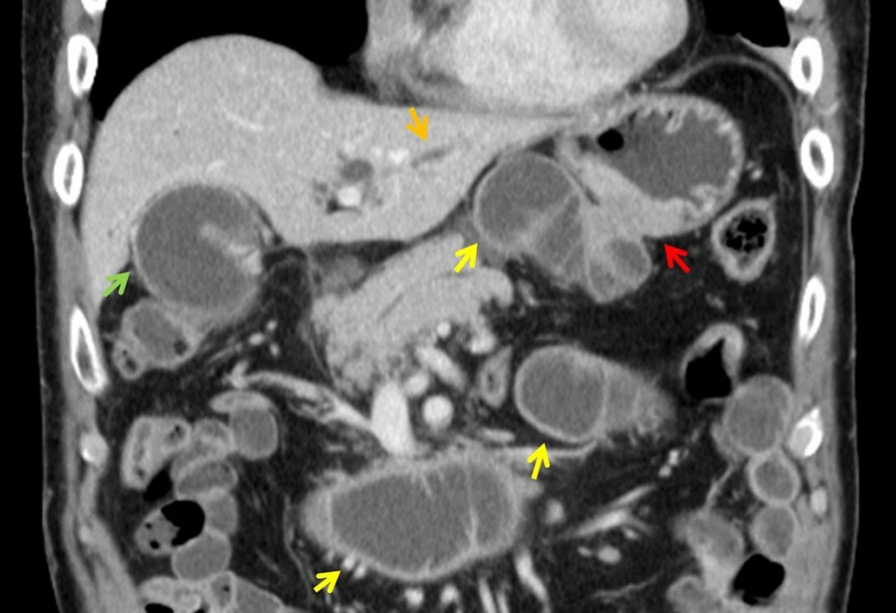
Abdominal contrast-enhanced computed tomography at initial presentation. The anastomosis site of the gastrojejunostomy (red arrow) was thickened. In addition to the afferent loop (yellow arrows), the common bile duct, bilateral intrahepatic bile ducts (orange arrow), and gallbladder (green arrow) were also significantly dilated

**Fig. 2:**
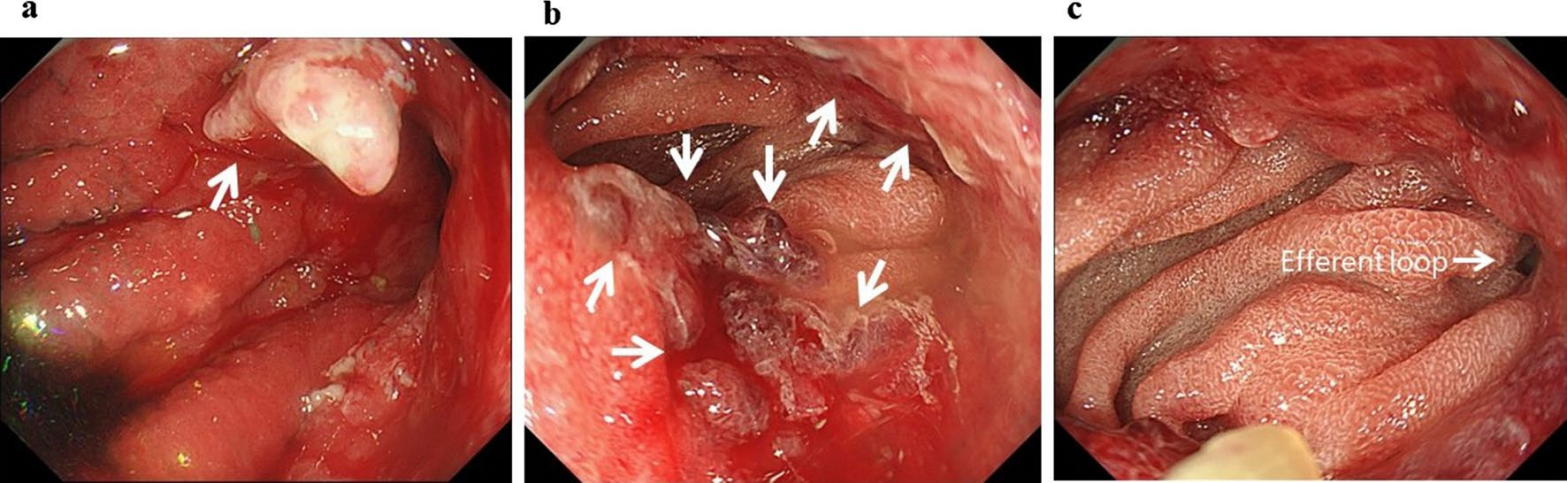
The pictures of the gastrointestinal fiberscope. **a** A 2-cm nodular lesion at the anterior wall of the stomach, just near the anastomosis site. **b** Irregular and hemorrhagic mucosa spread circularly from the nodular lesion to the anastomosed jejunum. **c** The efferent loop was patent

**Fig. 3:**
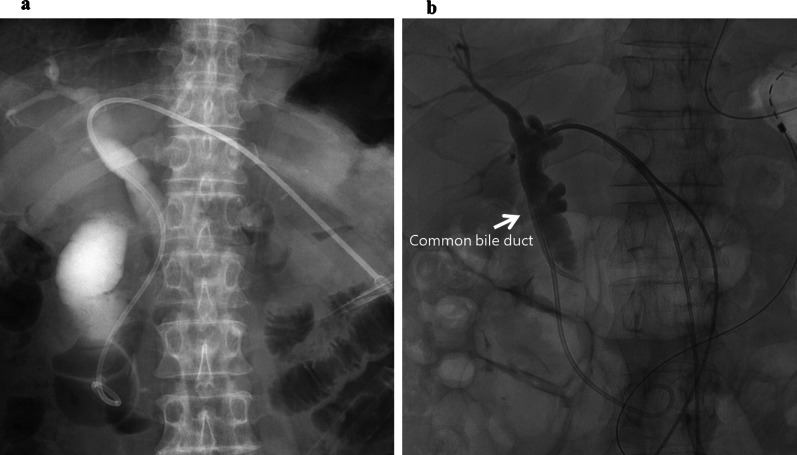
The images of the percutaneous transhepatic cholangial drainage. **a** An image of 1st PTCD. An 8.5-French pigtail catheter was inserted via the lateral inferior branch of the intrahepatic bile duct, with the catheter tip placed in the duodenum. **b** An image of 2nd PTCD. 8.5-French multiside hole pigtail catheter and 7-French multiside hole straight catheter were inserted into the duodenum and common bile duct, respectively

The physical examination was unremarkable. Laboratory tests revealed slightly elevated C-reactive protein (1.28 mg/dL) with no signs of pancreatitis, cholangitis (total amylase: 119 U/L, total bilirubin: 1.0 mg/dL, alkaline phosphatase: 283 U/L, aspartate aminotransferase: 21 UL, alanine aminotransferase: 14 U/L; all were at normal level). γ-Glutamyl transpeptidase was slightly elevated (69 U/L). The tumor markers, carbohydrate antigen 19-9 and carbohydrate antigen 125 were also elevated (310 U/ml and 37.0 U/mL, respectively). Abdominal contrast-enhanced computed tomography revealed thickening of the anastomosis site of the gastrojejunostomy as well as significant dilation of the afferent loop, common bile duct, bilateral intrahepatic bile ducts, and gallbladder (Fig. 1). Gastrointestinal fiberscopy revealed a 2-cm nodular lesion at the anterior wall of the stomach just near the anastomosis site (Fig. 2a). Irregular and hemorrhagic mucosa spread circularly from the nodular lesion to the anastomosed jejunum, which was suspected to be the spread of the tumor (Fig. 2b). The stoma of the afferent loop was not visible, indicating complete obstruction of the afferent loop. Thus, endoscopic decompression was not feasible. Meanwhile, the orifice of the efferent loop was patent (Fig. 2c). Based on these findings, the patient was clinically diagnosed with remnant gastric cancer with afferent loop syndrome. In order to prevent obstructive pancreatitis and cholangitis while providing time for preoperative scrutiny, a sonography-guided PTCD procedure was performed 3 days following the presentation. To decompress the afferent loop, an 8.5-French pigtail catheter was inserted via the lateral inferior branch of the intrahepatic bile duct, with the catheter tip placed in the duodenum (Fig. 3a).

Biopsy of the nodular lesion revealed a well-differentiated tubular adenocarcinoma. There were no sign of distant metastasis or peritoneal dissemination. Therefore, radical surgery was considered.

However, 18 days following the first PTCD, the patient developed a fever, and laboratory values revealed elevated total bilirubin (3.6 mg/dL), alkaline phosphatase (467 U/L), γ-glutamyl transpeptidase (230 U/L), total amylase (503 U/L), and transaminase (aspartate aminotransferase: 37 UL, alanine aminotransferase: 46 U/L). White blood cell count (9200/μL) and C-reactive protein level (14.87 mg/dL) were also elevated. On computed tomography, the afferent loop was still dilated, but to a lesser extent than the first PTCD. The patient was thought to have developed obstructive cholangitis and pancreatitis owing to insufficient decompression of the afferent loop. Since emergency surgery was considered risky in a situation where the patients had developed acute cholangitis and pancreatitis, sonography-guided PTCD was performed again. The former catheter placed in the duodenum was replaced by an 8.5-Fr multiside hole pigtail catheter, and a 7-French multiside hole straight catheter was added to the common bile duct via the same insertion point and intrahepatic bile duct (Fig. 3b). Defervescence was promptly achieved, and the serum total bilirubin, amylase, transaminase, C-reactive protein levels, and white blood cell count were normalized within a week from the second PTCD.

Although no signs of malnutrition were detected in the laboratory test results at presentation (serum albumin: 3.8 g/dL, total protein 7.3 g/dL, lymphocyte counts: 1500/μL; all were at normal level), the patient showed weight loss of 10% during the past one year; since it was considered to indicate malnutrition, we performed active nutrition therapy from the time of admission. Enteral nutrition was performed through a nasal feeding tube inserted into the efferent loop. The drainage of the PTCD tube was also administered via the feeding tube. Weight loss was not observed until the operation. Serum albumin level, total protein level, and lymphocyte counts had dropped at the operation (serum albumin: 2.8 g/dL, total protein: 5.8 g/dL, lymphocyte counts: 1100/μL), and it was thought to be owing to cholangitis and pancreatitis.

**Fig. 4:**
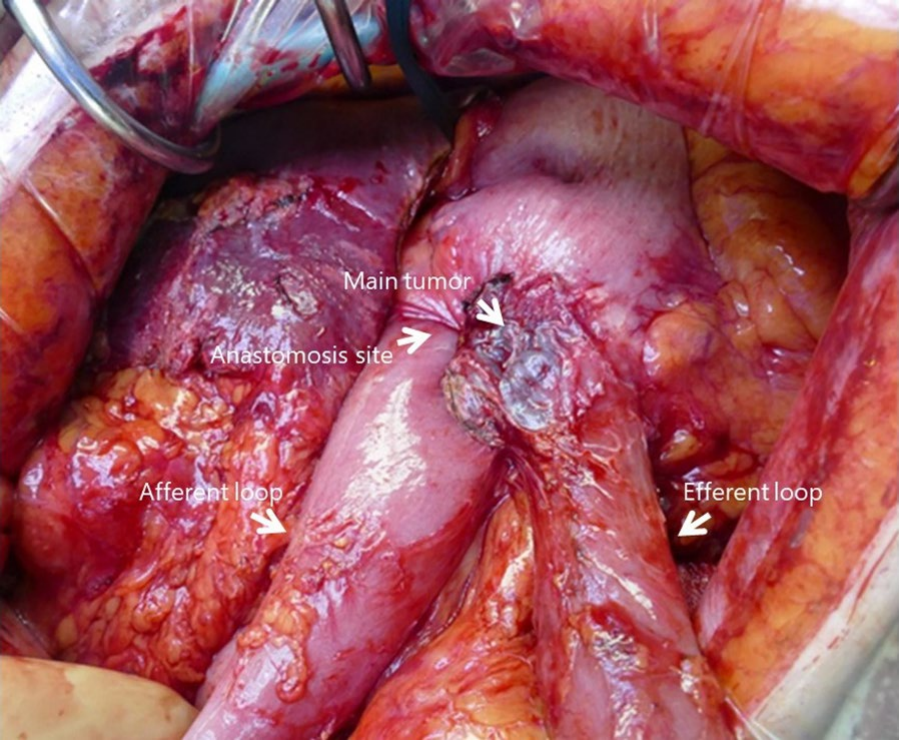
The picture in the operation. Reconstruction in the former operation was performed in antecolic Billroth II fashion without Braun’s anastomosis

After subsiding cholangitis and pancreatitis by second PTCD, an elective surgery was performed 10 days following the second PTCD. No signs of distant metastasis or peritoneal dissemination were observed during the operation, and the peritoneal lavage cytology was negative. The gastrojejunostomy of the former operation was performed via antecolic Billroth II without Braun’s anastomosis (Fig. 4). Total remnant gastrectomy, Roux-en-Y reconstruction, and jejunostomy were performed curatively. The patient developed intestinal obstruction due to bending of the jejunum at the jejunostomy site, but it was successfully managed with supportive therapy. The patient was discharged from the hospital on postoperative day 56th. Histopathological examination of the specimen revealed moderately differentiated tubular adenocarcinoma with invasion to the subserosal layer and no metastasis in the lymph nodes. All the surgical margins were intact. Postoperative adjuvant chemotherapy was not administered. The patient died 8 months following the operation owing to peritoneal dissemination.

## Discussion

Surgery is the cornerstone of treatment for afferent loop syndrome. Surgery consists of adhesiolysis and reconstruction for benign causes, and bypass or excision and reconstruction for malignant causes. In addition, endoscopic enteral stenting, PTCD, and direct percutaneous tube enterostomy play a principal role in the management of malignant cases as palliative therapy [5]. In most cases, the primary lesion is unresectable owing to local progression or distant metastasis. However, in our case, the primary lesion was resectable. PTCD was performed before surgery to allot time for the patient to recover, and control cholangitis and pancreatitis. In cases involving complete obstruction of the afferent loop, such as the present case, wherein endoscopic decompression was not feasible, the intrahepatic bile duct is dilated owing to accumulation of the biliary and pancreatic secretions. Therefore, sonography-guided PTCD is possible.

Even after PTCD, cholangitis and pancreatitis can still occur, as seen in this case. We thought this was owing to insufficient decompression of the afferent loop; however, this could be retrograde cholangitis and pancreatitis caused by dysfunction of the papilla of Vater owing to the first PTCD tube, which was inserted via the papilla of Vater. Besides, drainage for both afferent loop and common bile duct using multihole drainage tube could have been considered at the first PTCD.

Patients with chronic afferent loop syndrome tend to have poor nutrition owing to symptoms such as vomiting, nausea, and abdominal discomfort. Besides, bacterial overgrowth may result in malabsorption [5]. In such cases, the risk of emergency surgery increases. In this case, although the laboratory results did not suggest malnutrition, the patient presented a weight loss of 10% during the past year. Besides, preoperative enteral nutrition is recommended for patients undergoing upper major abdominal surgery independent of their nutrition risk [6]. Therefore, we actively managed his nutrition by enteral feeding through a nasal feeding tube inserted into the efferent loop. This method is viable for patients with efferent loop patents. We also dispensed the drainage liquid from the nasal feeding tube. This bile replacement following external drainage could benefit by not only improvement of the nutrition condition, but also preservation of the intestinal barrier function [7].

Cases of remnant gastric cancer with afferent loop syndrome are extremely rare. In a Japanese study including 101 cases of afferent loop syndrome, four cases (4.0%) were caused by remnant gastric cancer [8]. We searched the following sources for studies reporting cases of remnant gastric cancer with afferent loop syndrome: “remnant/residual gastric cancer” and “afferent loop syndrome/obstruction” (using PubMed). Only six cases were identified (Table 1). Our case was the first case in which elective surgery was performed following the management of afferent loop syndrome by PTCD. In previous cases as well as the present case, the onset time from the former operation was more than 30 years. Remnant gastric cancer is a differential diagnosis of afferent loop syndrome, especially in chronic cases with a later onset from the former operation. In all cases reported, reconstruction of the former gastrectomy was performed via Billroth II, and there were no cases involving Braun’s anastomosis.

**Table 1:** Reported cases of afferent loop syndrome caused by remnant gastric cancer

No.	Year	Fist author	Reason for gastrectomy	Reconstruction	Braun’s anastomosis	Onset time from the first operation	Obstruction of the afferent loop	Treatment	Postoperative complications	Prognosis (months, cause)	References
1	1984	Elyaderani	N/A	Billroth II	N/A	N/A	N/A	N/A	N/A	N/A	[9]
2	2001	Chevallier	Benign ulcer	Billroth II	N/A	31 years	Complete	–	–	Death (shortly after diagnosis, sepsis)	[10]
3	2006	Yazus	Benign ulcer	Billroth II	None	40 years	Incomplete	Operation	None	N/A	[11]
4	2013	Kawaoka	Gastric carcinoma	Billroth II	N/A	40 years	Incomplete	Operation	N/A	Death (4 months, recurrence)	[12]
5	2015	Sahin	Benign ulcer	Billroth II	None	47 years	Incomplete	Operation	Anastomotic leakage	N/A	[13]
6	2018	Cha	Benign ulcer	Billroth II	None	N/A	Incomplete	PTCD	–	Death (4 months, disease progression)	[14]
–	2021	Aoyama	Benign ulcer	Billroth II	None	43 years	Complete	PTCD → operation	Adhesive intestinal obstruction	Death (8 months, recurrence)	–

Our case was the first case in which elective surgery was performed after the management of afferent loop syndrome by PTCD. In many cases, the onset time of the former operation was more than 30 years

When distal gastrectomy is reconstructed via Billroth II, Braun’s anastomosis is typically supplemented to prevent postoperative bile reflux and alkaline reflux gastritis [15]. It can also prevent afferent loop syndrome caused by the obstruction of gastrojejunostomy. However, there are still cases of Billroth II reconstruction without Braun’s anastomosis [16]. The addition of Braun’s anastomosis to Billroth II reconstruction should be standardized to reduce the incidence of afferent loop syndrome caused by the obstruction of gastrojejunostomy.

We described the first case of remnant gastric cancer with afferent loop syndrome, which was successfully managed by performing elective surgery following management of the afferent loop syndrome by PTCD. In such cases, PTCD can be used not only to avoid emergency surgery, but also to maintain the patient’s nutritional condition. Cases of remnant gastric cancer are very rare, and all the reported cases occurred late following the first surgery.

## Conclusion

We presented a case of remnant gastric cancer with afferent loop syndrome, which was successfully treated by elective radical surgery following management of the afferent loop syndrome by PTCD. PTCD effectively managed afferent loop syndrome, resulting in safe elective surgery and the improvement of the patient’s condition. Remnant gastric cancer should be listed as a differential diagnosis of afferent loop syndrome, especially in chronic cases with a late-onset from the former operation.

## Data Availability

The authors declare that all the data in this article are available within the article.

## Abbreviation

PTCD: Percutaneous transhepatic cholangial drainage

## References

[1] Association JGC (2017). Japanese classification of gastric carcinoma (October 2017 [The 15th Edition]).

[2] Mak TK, Guan B, Peng J, Chong TH, Wang C, Huang S (2021). Prevalence and characteristic of gastric remnant cancer-a systematic review and meta-analysis. Asian J Surg.

[3] Tanigawa N, Nomura E, Lee SW, Kaminishi M, Sugiyama M, Aikou T (2010). Current state of gastric stump carcinoma in Japan: based on the results of a nationwide survey. World J Surg.

[4] Ballas KD, Rafailidis SE, Konstantinidis HD, Pavlidis TE, Marakis GN, Anagnostara E (2009). Acute afferent loop syndrome: a true emergency. A case report. Acta Chir Belg.

[5] Blouhos K, Boulas KA, Tsalis K, Hatzigeorgiadis A (2015). Management of afferent loop obstruction: reoperation or endoscopic and percutaneous interventions?. World J Gastrointest Surg.

[6] Kamiya S, Nagino M, Kanazawa H (2004). The value of bile replacement during external biliary drainage: an analysis of intestinal permeability, integrity, and microflora. Ann Surg.

[7] Weimann A, Braga M, Harsanyi L (2006). ESPEN guidelines on enteral nutrition: surgery including organ transplantation. Clin Nutr.

[8] Toyokawa T, Yamashita Y, Yamamoto A, Shimizu S, Inoue T, Nishiguchi Y (2014). Clinical evaluation of ten cases with afferent loop obstruction. J Abdom Emerg Med.

[9] Elyaderani MK, Gabriele OF (1984). Afferent loop syndrome: diagnostic features with new imaging methods. South Med J.

[10] Chevallier P, Gueyffier C, Souci J, Oddo F, Diaine B, Padovani B (2001). MRI of an afferent loop syndrome presenting as obstructive icterus. J Radiol.

[11] Yavuz N, Ergüney S, Ogüt G, Alver O (2006). Enteroliths developed in a chronically obstructed afferent loop coexisting with gastric remnant carcinoma: case report and review of the literature. J Gastroenterol Hepatol.

[12] Kawaoka T, Kuwahara T, Kaneko T, Harada T, Hiraki S, Fukuda S (2013). A case of remnant gastric cancer with afferent loop syndrome treated by left upper exenteration. Gan To Kagaku Ryoho.

[13] Şahin M, Ozlu B, Erdogan KE, Colak T (2015). Late onset remnant gastric cancer with afferent loop syndrome 47 years after Billroth II surgery. Case Rep Surg..

[14] Cha RR, Cho SB, Kim WS, Kim JJ, Lee JM, Lee SS (2018). Self-expanding metal stent procedure for afferent loop syndrome with ascending cholangitis caused by remnant gastric cancer: a case report. Medicine (Baltimore).

[15] Wang J, Wang Q, Dong J, Yang K, Ji S, Fan Y (2021). Total laparoscopic uncut Roux-en-Y for radical distal gastrectomy: an interim analysis of a randomized, controlled, clinical trial. Ann Surg Oncol.

[16] So JB, Rao J, Wong AS, Chan YH, Pang NQ, Tay AYL (2018). Roux-en-Y or Billroth II reconstruction after radical distal gastrectomy for gastric cancer: a multicenter randomized controlled trial. Ann Surg.

